# Supplementary material for: Carotenoid Cleavage Dioxygenase Genes of Chimonanthus praecox, CpCCD7 and CpCCD8, Regulate Shoot Branching in Arabidopsis
Source: Int J Mol Sci. 2021 Aug 15;22(16):8750. doi: 10.3390/ijms22168750 (PMC8395739; doi:10.3390/ijms22168750)
Supplement: Supplementary file 1 [file ijms-22-08750-s001.zip › ijms-1335244-supplementary.pdf]

```
1       ATGTCTCTCTCAGTGCCAGCCCACTTTCTTCCAACTAGACTCACAGGATGGGCTACAAAGAAGCCGGTGCAG
1        M  S  L  S  V  P  A  H  F  L  P  T  R  L  T  G  W  A  T  K  K  P  V  Q
73      AGAATCCCTCAGGCAAAAATAAATAGGGCAGTGTCAGGAACTGTGGGGGCGCCGGCAATCCACGTCCCTGAT
25       R  I  P  Q  A  K  I  N  R  A  V  S  G  T  V  G  A  P  A  I  H  V  P  D
145     GACGTCGACGTGGTTGAAGACGATGCGGTGGCTGCCTTTTGGGACTACCAGTTCTTGTTTGTCTCGCAGCGC
49       D  V  D  V  V  E  D  D  A  V  A  A  F  W  D  Y  Q  F  L  F  V  S  Q  R
217     ACCGAGTCCCGGGAACCCGTGGCGCTGCGGACTATTGAAGGAGCAGTCCCGTCGGACTTCCCTCGAGGAACC
73       T  E  S  R  E  P  V  A  L  R  T  I  E  G  A  V  P  S  D  F  P  R  G  T
289     TACTACCTGACCGGGCCCGGAATGTTCTCCGACGACTATGGGTCGACGGTGCACCCGCTTGACGGGCATGGC
97       Y  Y  L  T  G  P  G  M  F  S  D  D  Y  G  S  T  V  H  P  L  D  G  H  G
361     TACCTCAGGGCTTTTACAATTGACGGAATAACGGGAGAAGCCAGCTTCACAGCAAGGTACGTGGCAACGGAG
121      Y  L  R  A  F  T  I  D  G  I  T  G  E  A  S  F  T  A  R  Y  V  A  T  E
433     GCGCAGAAGGAGGAGTGGGATGGAGAGACCGGCAGGTGGAGGTTTACGCACCGGGGACCGTTTTCGGTGCTG
145      A  Q  K  E  E  W  D  G  E  T  G  R  W  R  F  T  H  R  G  P  F  S  V  L
505     AGGGGGGGGAGGAAGTTGGGGAACACGAAGGTGATGAAGAATGTGGCCAACACCAGTGTCTTGAGGTGGGGA
169      R  G  G  R  K  L  G  N  T  K  V  M  K  N  V  A  N  T  S  V  L  R  W  G
577     TCTCACCTCCTCTGCTTGTGGGAGGGCGGAGCCCCCTACCAGATTCATCCACGAACATTGGATACGATTGGC
193      S  H  L  L  C  L  W  E  G  G  A  P  Y  Q  I  H  P  R  T  L  D  T  I  G
649     CTGTTCGACGTGCTTGGAGGCGGTGGTGGTGGTGGCAAAGTGGAGGCAGAGGCGGTGGCGGAGGTGTCTCAG
217      L  F  D  V  L  G  G  G  G  G  G  K  V  E  A  E  A  V  A  E  V  S  Q
721     CTTTCAGATAGTCCGAGTAAGATTTCGGGAAGAGGGATCGTGGTTGCCGTCGCTGGCTTGTTGAAGCCGATT
241      L  S  D  S  P  S  K  I  S  G  R  G  I  V  V  A  V  A  G  L  L  K  P  I
793     CTGAACGGTGTATTCAAGATGCCTCCAAAGAGACTCTTGTCTCATTACAAGATAGATGCTCGAAGAAATCGA
265      L  N  G  V  F  K  M  P  P  K  R  L  L  S  H  Y  K  I  D  A  R  R  N  R
865     CTTCTTATGATCACTTGCAATGCTGAGGACATGCTTCTCCCTATTAGCAATTTTACTTTTCATGAATTTGAT
289      L  L  M  I  T  C  N  A  E  D  M  L  L  P  I  S  N  F  T  F  H  E  F  D
937     TTGAATTTCAAGTTACTACAAAAGCAGGAGTTTAGCATCCCTGACCATTTGATGATCCACGATTGGGCCTTC
313      L  N  F  K  L  L  Q  K  Q  E  F  S  I  P  D  H  L  M  I  H  D  W  A  F
1009    ACTGACACTCACTATATCATCTTTGGCAATCGGATCAAGCTTGATGTGCCTGGATCTTTGTTAGCAGTGTCT
337      T  D  T  H  Y  I  I  F  G  N  R  I  K  L  D  V  P  G  S  L  L  A  V  S
1081    GGACTATCTCCAATGATTTCTGCGTTGTCTGTAAATCCAAGCAAGCCCACATCACCCATTTATTTACTGCCA
361      G  L  S  P  M  I  S  A  L  S  V  N  P  S  K  P  T  S  P  I  Y  L  L  P
1153    AGATTTTCAAAGAAGAATCATATAAATAGAGAATGGCAAGTGCCACTGGAGGTTCCATCACAATTATGGTTG
385      R  F  S  K  K  N  H  I  N  R  E  W  Q  V  P  L  E  V  P  S  Q  L  W  L
1225    CTACATGTTGGCAATGCATTTGAGAGAAGGGATGATCAAGGAAACTTGGGAATTCAAATATATGCAGCTGCT
409      L  H  V  G  N  A  F  E  R  R  D  D  Q  G  N  L  G  I  Q  I  Y  A  A  A
1297    TGCTCCTATCAATGGTTCAACTTTCACAAAATGTTTGGATATGATTGGCAAAGTGGCAAACTAGATCCCTCA
433      C  S  Y  Q  W  F  N  F  H  K  M  F  G  Y  D  W  Q  S  G  K  L  D  P  S
1369    TTCATGAACGCAAGAGATGGTGAAGAGTCCTCGTTGCCTCATCTTGTTAAGCTATCTATTGAAGTAAATGCA
457      F  M  N  A  R  D  G  E  E  S  S  L  P  H  L  V  K  L  S  I  E  V  N  A
1441    AATGGAGTATGTGAGAAATGTTCAGTAGATCCCTTAAATCAATGGCACAGACCATCGGATTTCCCTGCCATC
481      N  G  V  C  E  K  C  S  V  D  P  L  N  Q  W  H  R  P  S  D  F  P  A  I
1513    AATCCAGCCTTCTCCGGTGGCAAAAACAAATACATCTACGCGGCGACAACTTCAGGATCTCGACAGAACTTA
505      N  P  A  F  S  G  G  K  N  K  Y  I  Y  A  A  T  T  S  G  S  R  Q  N  L
1585    CCGCATTTTCCTTTTGATAGTGTGGCAAAGATCAACATCTCCGATGGGTCAGTATCAACATGGTCCTCCAGA
529      P  H  F  P  F  D  S  V  A  K  I  N  I  S  D  G  S  V  S  T  W  S  S  R
1657    AGCCGACGATTCATCGGTGAGCCTGTCTTCATTCCCAAAGGCGGGGACGAGGATGACGGATACCTTCTCGTT
553      S  R  R  F  I  G  E  P  V  F  I  P  K  G  G  D  E  D  D  G  Y  L  L  V
1729    GTAGAGTATGCAGTGTCAATACAGAGATGTTATCTGGTGATTTTGGATCCGAAACGCATAGGAAAAGCTGGA
577      V  E  Y  A  V  S  I  Q  R  C  Y  L  V  I  L  D  P  K  R  I  G  K  A  G
1801    GCAGTTGTGGCAAAGCTGGAAGTTCCCAAACAGCTCAATTTTCCACTCGGATTCCATGGAATTTGGGCACCA
601      A  V  V  A  K  L  E  V  P  K  Q  L  N  F  P  L  G  F  H  G  I  W  A  P
1873    CATTAG
625      H  *
```

Figure S1. Nucleotide and amino acid sequence of *CpCCD7*

```
1       ATGGCTTCTCTATCAGTCTCTGTTGCCGGAAAAAGTATCTTTCCGGCTACCTCATCGGATTCATTTGAATCC
1        M  A  S  L  S  V  S  V  A  G  K  S  I  F  P  A  T  S  S  D  S  F  E  S
73      GATCGTCGAGGGAATGCTTCTCTCTCAATGGGTCCATCACCAATGGCGCGTCGCATGCTAGGCTCACATCTT
25       D  R  R  G  N  A  S  L  S  M  G  P  S  P  M  A  R  R  M  L  G  S  H  L
145     GTGAGCGTCGCAACCCGGCCGTCGATCATTGCTCCGTCTGAAACAATGGAGACGTACGATGGAAAGAAACTA
49       V  S  V  A  T  R  P  S  I  I  A  P  S  E  T  M  E  T  Y  D  G  K  K  L
217     GCAGCATGGAAGAGCATTCAAAATGAAAGATGGGAAGGAGAGCTCGACGTTGAAGGCGAAATTCCATTATGG
73       A  A  W  K  S  I  Q  N  E  R  W  E  G  E  L  D  V  E  G  E  I  P  L  W
289     CTGAAAGGCACATATCTAAGGAATGGGCCTGGGCTTTGGAACATTGGAGACTATAATTTCCGCCACCTCTTC
97       L  K  G  T  Y  L  R  N  G  P  G  L  W  N  I  G  D  Y  N  F  R  H  L  F
361     GATGGCTATGCAACCCTTGTCCGCCTCCACTTTGAAAATGGCCGGCTGGTGGCAGGCCACCGGCAAATTGAA
121      D  G  Y  A  T  L  V  R  L  H  F  E  N  G  R  L  V  A  G  H  R  Q  I  E
433     TCACAAGCATACAAGGCTGCAATGAAGAACAACAAGCTATGCTACAGAGAATTTTCCGAGGCGCCGAAGATG
145      S  Q  A  Y  K  A  A  M  K  N  N  K  L  C  Y  R  E  F  S  E  A  P  K  M
505     GACAATTTTCTAGCGTATATGGGTGAGCTGGCCAGCCTCTTCTCCGGCGCATCGCTGACTGATAATGCCAAC
169      D  N  F  L  A  Y  M  G  E  L  A  S  L  F  S  G  A  S  L  T  D  N  A  N
577     ACCGGTGTCGTTAGGCTTGGGGATGGCCGGGTTGTCTGCTTGACGGAGACAATCAAGGGCTCGATCGAAGTG
193      T  G  V  V  R  L  G  D  G  R  V  V  C  L  T  E  T  I  K  G  S  I  E  V
649     GATCCAAACACATTAGAAACAATTGGTAAGTTTGAGTACACGGACGAATTAGGTGGGCTGATCCACTCGGCC
217      D  P  N  T  L  E  T  I  G  K  F  E  Y  T  D  E  L  G  G  L  I  H  S  A
721     CACCCTATTGTGACGGGCTCAGAGTTCTTGACACTTCTGCCAGACTTGGTGAGGCCCGGGTACACTGTGGTC
241      H  P  I  V  T  G  S  E  F  L  T  L  L  P  D  L  V  R  P  G  Y  T  V  V
793     CGGATGGAGCCCAATAGTAATGAAAGGAAGGTGATTGGAAGGGTGGACTGCCGGGGTGGGCCGGCCCCTGGG
265      R  M  E  P  N  S  N  E  R  K  V  I  G  R  V  D  C  R  G  G  P  A  P  G
865     TGGGTCCATTCATTCCCTGTGACTGAGCACTATATCATTGTGCCAGAGATGCCACTGAGGTATTGCGCCCAG
289      W  V  H  S  F  P  V  T  E  H  Y  I  I  V  P  E  M  P  L  R  Y  C  A  Q
937     AATCTGCTAAGGGCTGAGCCCACACCATTGTACAAGTTTGAGTGGCACCCTCACTCCAAAGCATTCATGCAT
313      N  L  L  R  A  E  P  T  P  L  Y  K  F  E  W  H  P  H  S  K  A  F  M  H
1009    GTGGTCTGTAGAGCCAGTGGAAAGATTGTGGCAAGTGTGGAGGTTCCATTATTTGTGACATTCCATTTCATC
337      V  V  C  R  A  S  G  K  I  V  A  S  V  E  V  P  L  F  V  T  F  H  F  I
1081    AATGCCTACGAGGAGAAAGATGAGGATGGAAGGGTGACGGCGATCATAGCCGACTGTTGCGAGCACAATGCC
361      N  A  Y  E  E  K  D  E  D  G  R  V  T  A  I  I  A  D  C  C  E  H  N  A
1153    GACGCCACCATACTCGACAAGCTTCGGCTGCAAAATCTCCGGTCGTTTTCCGGCGAAGACGTATTGCCCGAT
385      D  A  T  I  L  D  K  L  R  L  Q  N  L  R  S  F  S  G  E  D  V  L  P  D
1225    GCTAAAGTTGGGCGTTTTATAATTCCATTGGATGGAAGTCCTAAAGGAAAGCTAGTGGCAGCATTGGAGCCT
409      A  K  V  G  R  F  I  I  P  L  D  G  S  P  K  G  K  L  V  A  A  L  E  P
1297    GAGCAACATGGGAAAGGCATGGATATGTGCAGTATCAACCCTGCCTACTTAGGAAAGAAGTACAGATACGCT
433      E  Q  H  G  K  G  M  D  M  C  S  I  N  P  A  Y  L  G  K  K  Y  R  Y  A
1369    TATGCTTGCGGAGCCCAACGCCCCTGCAACTTTCCCAACACCATTACAAAGATCGATTTGGTGGAGAAGAAA
457      Y  A  C  G  A  Q  R  P  C  N  F  P  N  T  I  T  K  I  D  L  V  E  K  K
1441    GCAAAGAACTGGCATGATGAGGGAGCTGTACCCTCCGAGCCCTTCTTCGTAGCTAGGCCTGGTGCAACTGAA
481      A  K  N  W  H  D  E  G  A  V  P  S  E  P  F  F  V  A  R  P  G  A  T  E
1513    GAAGATGATGGTGTTGTAATTTCCATGATTAGTGACAAAAATGGAGAAGGCTATGCATTGTTGTTGGATGGA
505      E  D  D  G  V  V  I  S  M  I  S  D  K  N  G  E  G  Y  A  L  L  L  D  G
1585    TCCACATTCGAAGAACTTGCAAGAGCAAAGTTCCCATATGGTCTTCCCTATGGTCTCCATGGATGTTGGGTT
529      S  T  F  E  E  L  A  R  A  K  F  P  Y  G  L  P  Y  G  L  H  G  C  W  V
1657    CCAAAGAATTAA
553      P  K  N  *
```

Figure S2. Nucleotide and amino acid sequence of *CpCCD8*

Table S1. Primers used for *CpCCD7* and *CpCCD8* PCR and quantitative real-time PCR (qRT-PCR)

Primer sequences (5'-3')	Primer sequence (5'-3')	Purpose of primers	Annealing temperatures℃
CpCCD7-F	GATCTCTCGATATTATATCGTGAGTT	Full-length amplification	56
CpCCD7-R	TACAAGTGATTATGATTTCCATTATC		
CpCCD8-F	CATTCTGGCCATGGCTTCTCTATC		56
CpCCD8-R	CATCCATGCTCATCATCATTGTCG		
qRT-CpCCD7-F	GGAATGTTCTCCGACGACTATGG	Real-time quantification	58
qRT-CpCCD7-R	CATCACCTTCGTGTTCCCCAAC		
qRT-CpCCD8-F	CGTATTGCCCGATGCTAAAGTTG		58
qRT-CpCCD8-R	TCCGCAAGCATAAGCGTATCTGT		

CpActin-F	AGGCTAAGATTCAAGACAAGG	58
CpActin-R	TTGGTCGCAGCTGATTGCTGTG	
CpTublin-F	GTGCATCTCTATCCACATCG	58
CpTublin-R	CAAGCTTCCTTATGCGATCC	
qRT-AtBRC1-F	TTCCCAGTGATTAACCACCAT	56
qRT-AtBRC1-R	TCCGTAAACTGATGCTGCTC	
AtActin-F	GACTCAGATCATGTTTGAGACCTTT	59
AtActin-R	CCAGAGTCCAACACAATACCG	

Table S2. Primers used for plasmid construction

Primer name	Primer sequences (5'-3')	Purpose of primers	Annealing temperatures°C
CpCCD7-F	GGGGACAAGTTTGTACAAAAAAGCAGGCT *att*B1 TATCAGATGTCTCTCTCAGTGCCAGC	PGWB551	66
CpCCD7-R	GGGGACCACTTTGTACAAGAAAGCTGGGT *att*B2 TACAAGTGATTATGATTTCCATTATC		
CpCCD8-F	GGGGACAAGTTTGTACAAAAAAGCAGGCT *att*B1 ATGGCTTCTCTATCAGTCTCTGTTG		65
CpCCD8-R	GGGGACCACTTTGTACAAGAAAGCTGGGT *att*B2 ATTCTTTGGAACCCAACATCCATGG		
CpCCD7-F	GGGGTACC TATCAGATGTCTCTCTCAGTGCCAG	pCAMBIA 1300	63.5
CpCCD7-R	GCTCTAGA ATGTGGTGCCCAAATTCCATGGAAT		
CpCCD8-F	GGGGTACC CATTCTGGCCATGGCTTCTCTATC		63.5
CpCCD8-R	GCTCTAGA ATTCTTTGGAACCCAACATCCATG		
